# Code-based refinement and finite element validation of punching shear design for reinforced concrete footings

**DOI:** 10.1038/s41598-026-68850-7

**Published:** 2026-09-09

**Authors:** Dina M. Ors, Nada M. Abdelhamid, Amr H. Zaher, Ahmed M. Ebid

**Affiliations:** 1 Structural and Construction Management Department, Future University in Egypt (FUE), Cairo, Egypt; 2 Structural Engineering Department, Faculty of Engineering, Ain Shams University, Cairo, Egypt

**Keywords:** Punching shear, Reinforced concrete, Isolated footings, Finite element modeling, Soil–structure interaction, Design codes, Engineering, Materials science

## Abstract

Punching shear failure of reinforced concrete (RC) footings is strongly influenced by soil–structure interaction; however, current design codes evaluate footing punching capacity using slab-based empirical formulations. This study presents a code-based refinement and Finite Element Model (FEM) validation of punching shear design for reinforced concrete (RC) isolated footings resting on soil. A three-dimensional nonlinear Finite Element Model (FEM) was developed in PLAXIS 3D to simulate concrete cracking and crushing, Reinforcement yielding, soil plasticity, and concrete–soil interface behavior. The model was validated against experimental results from nine square footings (750 × 750 × 120 mm) tested under monotonic vertical loading. Numerical predictions of ultimate punching load and load–settlement response agreed well with experiments, with discrepancies limited to ± 10% and coefficients of variation not exceeding 1.11%. A comprehensive parametric study comprising 81 finite element models was then conducted to investigate the effects of concrete compressive strength (34, 42, and 61 MPa), column aspect ratio (a/b = 1, 2, and 3), punching shear Reinforcement (none, 8Ø6, and 8Ø8), and soil stiffness (sand, sand–crushed limestone mixture, and crushed limestone). Results showed that increasing concrete strength enhanced punching capacity by up to 34%, while increasing column aspect ratio increased capacity by up to 74%. Soil stiffness was identified as a governing parameter, increasing punching capacity by up to 20%, subgrade reaction by up to 200%, and reducing settlement by up to 75%. ACI 318 and ECP 203 predictions exhibited significant scatter when directly applied to footings. A refined punching shear expression was therefore proposed by introducing a correction factor derived from the finite element results. The soil-related factor (η₃ = ln(5000kₛ)) was found to dominate, while other correction factors remained unity within the investigated ranges. The proposed formulation significantly improves the reliability of punching shear design for RC footings by explicitly accounting for soil stiffness effects.

**Supplementary Information:**

The online version contains supplementary material available at https://doi.org/10.1038/s41598-026-68850-7.

## Introduction

Punching shear failure of reinforced concrete (RC) footings is a brittle and potentially catastrophic mode of failure that occurs when concentrated column loads cause a truncated-cone separation of concrete around the column–footing interface. Unlike slab–column connections, RC footings are directly supported by soil, which introduces upward contact pressure, nonuniform stress distribution, and soil–structure interaction effects that significantly influence the internal force transfer mechanism. Despite these fundamental differences, current design practice does not provide a separate equation for footings and designers have to evaluate the punching shear resistance of footings using slab-based empirical equations, which were originally calibrated against experimental databases of flat slabs rather than soil-supported foundations. Major international design codes, including ACI 318, Eurocode 2, and fib Model Code 2010^1–3^, adopt simplified punching shear formulations based on a critical control perimeter and concrete shear strength^4,5^. While these provisions provide reasonable predictions for slab-column systems, several studies have shown that their direct application to footings leads to significant scatter, excessive conservatism, or unconservative estimates, depending on footing geometry and support conditions^6–8^. Experimental evidence consistently indicates that soil support modifies stress trajectories, delays crack localization and affects the punching failure mechanism compared to suspended slabs. In response to these limitations, researchers have increasingly relied on finite element modeling (FEM) to capture the nonlinear behavior of RC footings, including cracking, crushing, reinforcement yielding, and soil–structure interaction. When properly calibrated, finite element models can accurately reproduce experimental load–displacement responses and failure loads, making them a reliable tool for parametric studies and design-oriented investigations^9–13^. However, despite growing numerical research, validated Finite Element-based refinements of code equations specifically tailored for footings remain limited. Accordingly, this study aims to validate a nonlinear finite element model for RC footings against available experimental results, conduct a comprehensive parametric study, and propose a code-based refinement that enables slab-derived punching shear equations to be applied more reliably to RC footings.

Punching shear design provisions in most codes are empirical and largely derived from slab–column connection tests. In ACI 318, the punching resistance is evaluated along a control perimeter located at distance equal to half the slab depth (*d/2)* from the column face, assuming uniform shear distribution and neglecting support conditions beneath the slab^1^. Eurocode 2 and fib Model Code 2010 adopt similar perimeter-based approaches, with modifications accounting for reinforcement ratio and size effect^2,3^. When applied to footings, these formulations implicitly assume that punching resistance is governed solely by concrete strength and geometry, without accounting for the beneficial contribution of upward soil reaction. This simplification has been shown to produce inconsistent predictions, particularly for footings with small shear span-to-depth ratios (*a/d*)^14^.

Siburg and Hegger^15^ carried out an extensive experimental program to examine the punching shear response of reinforced concrete footings subjected to uniform soil pressure. Their study included thirteen large-scale specimens with realistic dimensions, featuring footing plan sizes ranging from 1.20 × 1.20 m to 2.70 × 2.70 m, thicknesses between 0.45 and 0.65 m, and shear span-to-effective depth ratios of approximately 1.25 to 2.00. These tests were complemented by earlier experimental work conducted at Rhenish-Westphalian Technical University, resulting in a total database of twenty-two tested footings. The experimental campaign focused on evaluating the effects of effective depth, concrete compressive strength, flexural reinforcement ratio, and the use of punching shear reinforcement. For footings without shear reinforcement, the results demonstrated that the punching shear capacity is highly sensitive to the shear span-to-depth ratio, with increasing ratios leading to a noticeable reduction in resistance. This trend was consistently observed across different concrete strength levels, reinforcement ratios, and footing thicknesses. In addition, the measured punching capacities were found to correlate with the cubic root of the concrete compressive strength, $\sqrt[3]{{f}_{ck}}$. The depth-dependent size effect factor k adopted in Eurocode 2 was shown to underestimate the influence of member depth, suggesting that further experimental verification is required for thicker footing configurations. For footings incorporating vertical shear reinforcement, the effectiveness of the reinforcement was influenced by the shear span-to-depth ratio. Although reducing the distance between the first stirrup row and the column face did not lead to a significant increase in punching resistance, it resulted in a more uniform cracking pattern. In contrast, stirrup spacing greater than 0.3d was associated with a decrease in punching shear capacity.

Several experimental investigations have highlighted the inadequacy of slab-based punching shear equations for footings. Vacev et al.^9^ conducted experimental testing and finite element analysis of RC column footings and reported that measured punching capacities often exceeded ACI predictions^1^, especially for stocky footings. The authors attributed this discrepancy to the altered stress field caused by soil support. A comprehensive evaluation by Santos et al^14^. analyzed 216 experimental tests on RC footings, comparing predictions from ACI 318^1^, Eurocode 2^2^, and Brazilian code provisions^16^. The study reported large scatter in experimental-to-predicted strength ratios and identified the shear span to depth ratio (*a/d)* as a critical parameter insufficiently represented in current codes. Other experimental studies have investigated the effects of reinforcement ratio, footing thickness, and load eccentricity, consistently confirming that footings exhibit a distinct punching shear behavior compared to slabs, even when geometrically similar^11,12,17,18^.

Finite element modeling has become an essential tool for studying punching shear in RC footings due to its ability to simulate three-dimensional stress states and nonlinear material behavior^19^. Vacev et al.^9^ demonstrated that three-dimensional nonlinear finite element models could accurately reproduce cracking patterns, load–settlement behavior, and ultimate punching loads observed experimentally. Ahmed et al.^20^ further showed that Finite Element-based predictions of punching capacity are generally closer to experimental results than code-based equations, particularly when soil support is explicitly modeled. More recent numerical studies have extended finite element analyses to parametric investigations, examining the influence of footing geometry, reinforcement ratio, column size, and soil stiffness^11,21–23^. These studies emphasize that validated finite element models provide a robust framework for identifying correction factors that can enhance the accuracy of existing design equations.

## Research gap

Based on the reviewed literature, the following gaps have been identified: existing punching shear design codes are primarily slab-oriented and do not provide footing-specific formulations; experimental evidence shows that soil–structure interaction significantly influences punching behavior; and although finite element modeling has been widely used for validation, few studies have translated validated numerical results into refined, code-based design expressions.

## Objective

**Fig. 1:**
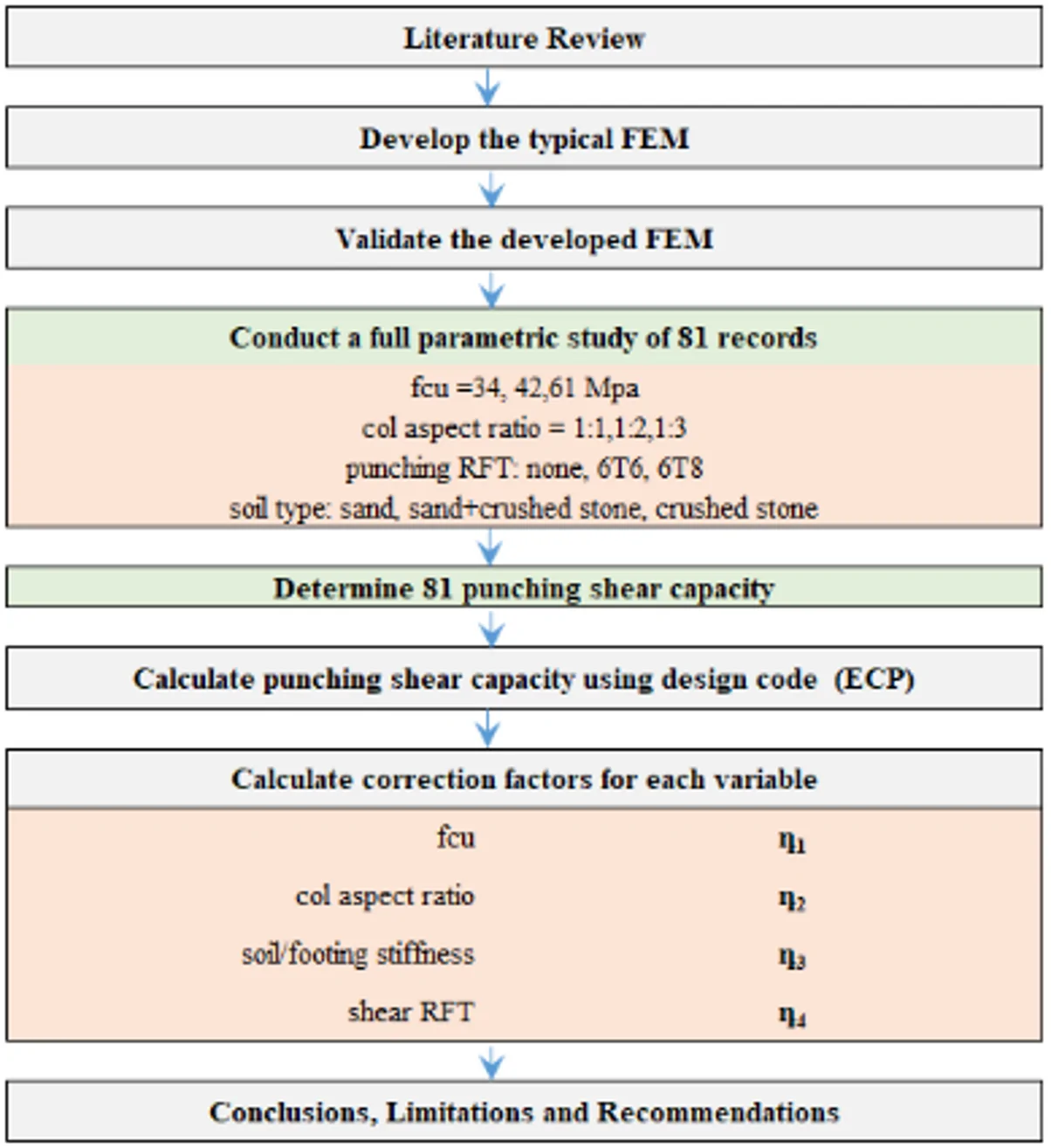
The applied methodology

The present study addresses the research gaps by integrating experimental validation, finite element parametric analysis, and direct refinement of punching shear design equations for RC footings. The design codes considered in this study are the ECP^24^ and ACI 318^1^. To achieve this objective, a nonlinear finite element model for RC footings is developed and validated against comprehensive experimental data. Then, parametric study using the validated finite element model is conducted to quantify the influence of key design variables on the punching shear capacity of the RC footings rested on different soil types. Finally, correction factor is applied to enhance the available design equations with the aim of improving accuracy and reliability for footing applications rather than slab analogies alone (Fig. 1).

## Reference experimental investigation

**Fig. 2:**
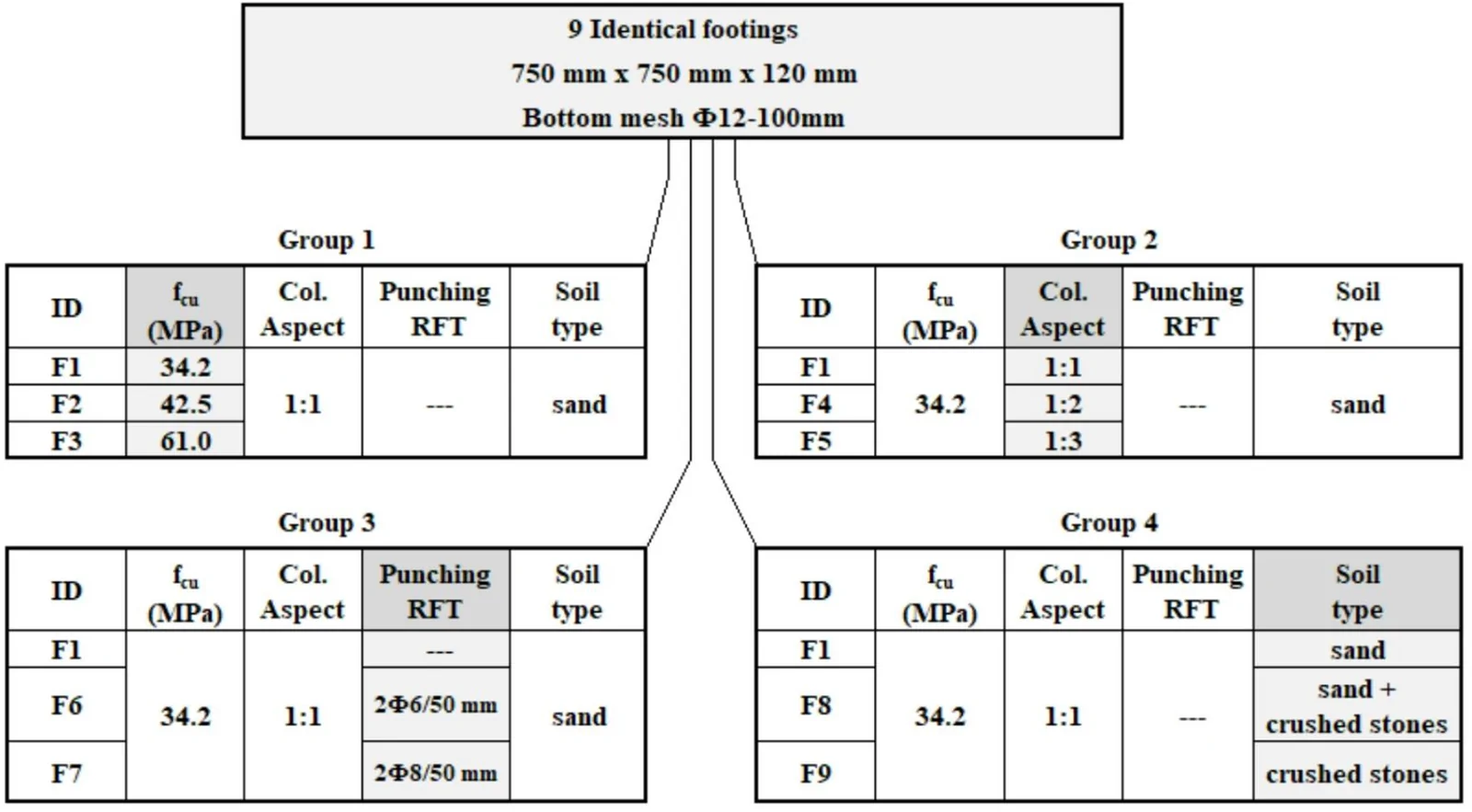
Grouping of all Tested reinforced concrete Footings^25,26^.

**Fig. 3:**
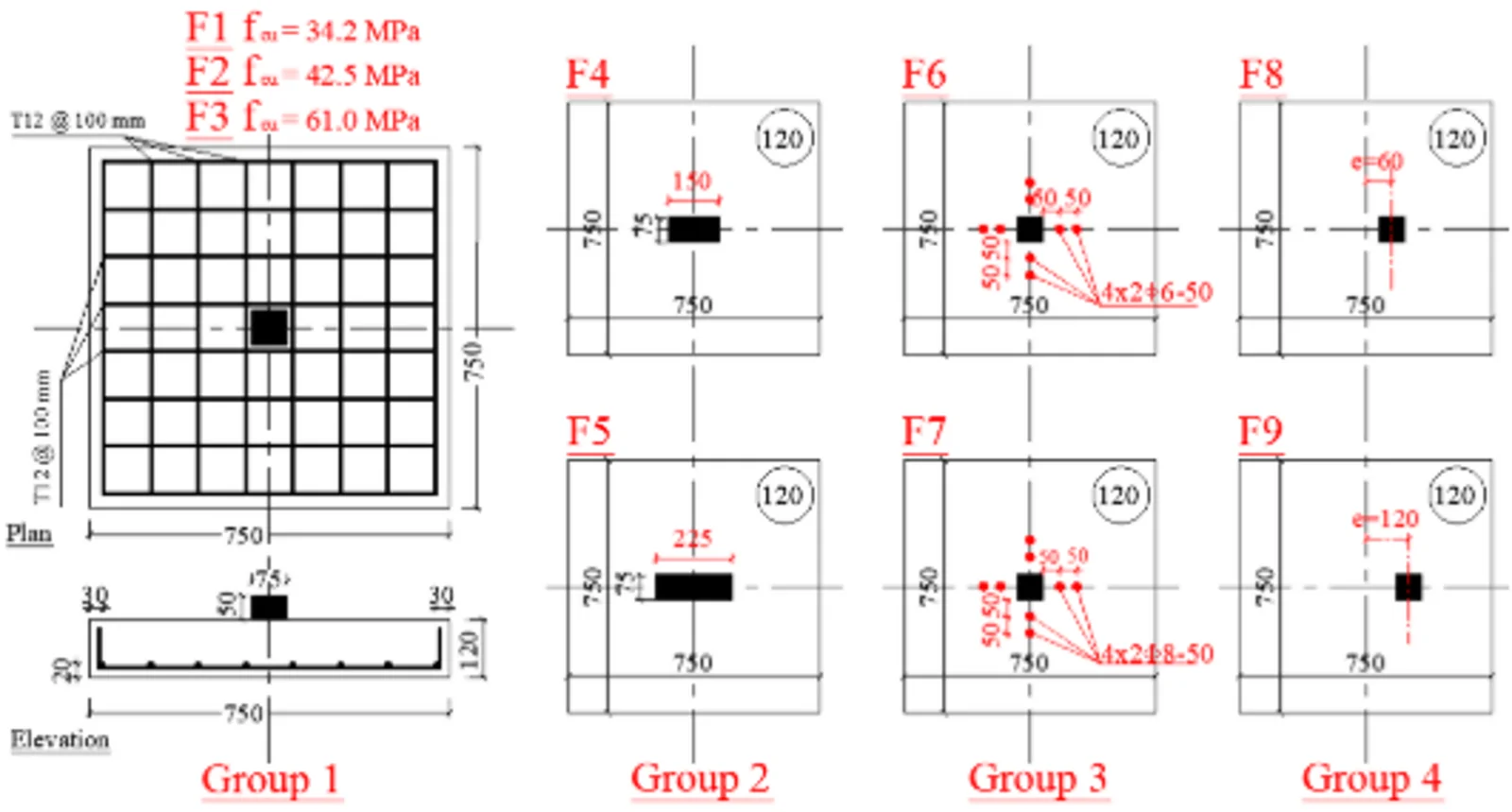
Configurations of All Tested Footing Specimens. (all dimensions are in mm)

Total of nine square footings designated as F1 through F9, each measuring 750 mm × 750 mm in plan and constant thickness of 120 mm, were fabricated and experimentally tested under incrementally applied vertical static loading until failure. All footings were reinforced with a bottom reinforcement mesh of Ø12 bars spaced at 100 mm in both directions. The investigated variables included concrete compressive strength (${f}_{cu}$), column aspect ratio (a:b), and punching shear reinforcement. Each variable constituted a group of three footings, to assess the influence of each parameter on the punching shear behavior of the tested footings under identical loading conditions. The details of the experimental matrix are presented in Fig. 2, which illustrates the specimen grouping, and Fig. 3, which provides detailed drawings of the tested specimens. A summary of the experimental program is presented herein, while the complete details of the experimental work, including specimen preparation, test setup, instrumentation, and testing procedures, are presented in the authors' previous publications^25,26^.

## Typical finite element model

### Concrete

**Fig. 4:**
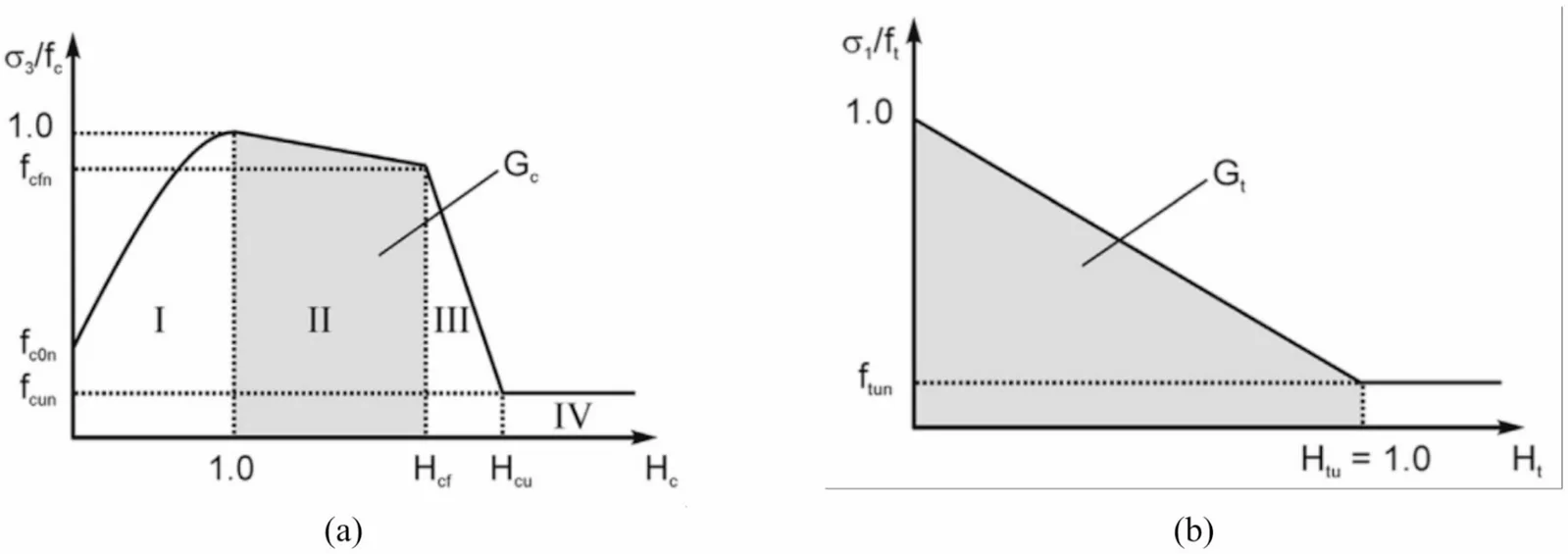
Normalized stress–strain curve in compression and tension^31^.

The concrete model in PLAXIS is formulated to represent the nonlinear behavior of concrete and concrete-like materials, incorporating tensile cracking, strain softening, and time-dependent effects. Originally developed for shotcrete applications in tunneling^27^, it has since been applied to jet-grouted slabs and mass concrete elements^28,29^. The model adopts a fracture energy-based approach to capture post-peak tensile behavior. Its formulation is conceptually related to bonded material frameworks derived from Structured Cam Clay and CASM, and shares similarities with the Concrete Damaged Plasticity (CDP) model, with key differences in yield surface definition and the ability to account for creep and shrinkage^30^. Under compressive loading, the Concrete constitutive model adopts the formulation proposed by Schütz, Potts, and Zdravković^31^. The uniaxial stress–strain response is discretized into four computational segments, as shown in Fig. 4a: (I) quadratic hardening up to peak stress, (II) linear post-peak softening, (III) continued linear softening, and (IV) a constant residual-strength branch to ensure numerical stability. To capture the time-dependent evolution of material behavior within the Finite Element Model framework, a normalized hardening–softening parameter ${H}_{c}={\varepsilon}_{p3}/{\varepsilon}_{pcp}$ is employed, where ${\varepsilon}_{p3}$ is the minor plastic strain derived from the compressive strength ${F}_{c}$, and ${\varepsilon}_{pcp}$ corresponds to the plastic strain at peak stress in uniaxial compression. Table 1 summarizes the material input parameters used for the different concrete constitutive models for each concrete grade considered in this study.

**Table 1:** Concrete material input parameters implemented in PLAXIS for all analyzed footings.

Parameter				Value		
Description		Symbol	Unit	f_cu_ = 34MPa	f_cu_ = 42MPa	f_cu_ = 61MPa
Density		γ	kN/m^3^	25	25	25
Compression	Young’s modulus	E₂₈	MPa	23,000	25,700	36,300
	Poisson’s ratio	ν	–	0.20	0.20	0.20
	Uniaxial compressive strength	f_c,28_	kPa	28,000	34,000	50,000
	Normalized initially mobilized strength	f_c0n_	–	0.39	0.39	0.39
	Normalized failure strength	f_cfn_	–	**0.78**	**0.76**	**0.75**
	Normalized residual strength	f_cun_	–	0.01	0.01	0.01
	Compressive fracture energy	G_c,28_	kN/m	0.05	0.04	0.03
	Maximum friction angle	φₘₐₓ	°	37	37	37
	Dilatancy angle	Ψ	°	4	4	4
Tension	Safety factor for compressive strength	γ_fc_	–	1	1	1
	Uniaxial tensile strength	f_t,28_	kPa	2800	3400	4000
	Ratio of residual to peak tensile strength	f_tun_	–	0.01	0.01	0.01
	Tensile fracture energy	G_t,28_	kN/m	0.05	0.11	**0.15**
	Safety factor for tensile strength	γ_ft_	–	1	1	1

### Steel

The steel reinforcement in the concrete footing was explicitly modeled in PLAXIS using embedded beam elements, ensuring full compatibility with the surrounding concrete continuum. The beams were positioned according to the same layout and spacing used in the experimental program, providing an accurate representation of the reinforcement arrangement and allowing direct comparison between numerical and experimental results. The reinforcing steel was assigned an elasto-plastic constitutive behavior, exhibiting linear elastic response up to yielding followed by plastic deformation, to realistically capture the nonlinear response of the bars under increasing load. In the PLAXIS material model, the steel was defined by Young’s modulus and yield strength while the cross-sectional area and moments of inertia in both the second and third directions were defined based on the bar diameter as summarized in Table 2. The cross-sectional area governs axial stiffness, and the moments of inertia control the bending stiffness of the embedded beams. Different sets of material and sectional properties were applied to reflect the variation in steel strength grades for the high tensile steel in the main reinforcement and the punching-shear mild reinforcement, as well as the bar diameters used in the experimental program, ensuring that the numerical model accurately reproduces the mechanical response of each reinforcement configuration.

**Table 2:** Parameters defined in PLAXIS for the elastic-perfect plastic material model of steel reinforcement.

Parameter			Value		
Description	Symbol	Unit	Main reinforcement	Punching reinforcement	
Density	γ	kN/m^3^	87.50	87.50	
Young’s modulus in axial direction	E	MPa	2 *×* 10^8^	2 *×* 10^8^	
Yield strength	σ_y_	MPa	400	240	
Rebar diameter	Ø	mm	12	8	6

### Soil

The soil materials were modeled using the Mohr—Coulomb constitutive model available in PLAXIS, which captures both the elastic and plastic behavior of granular materials under loading. As reported in the references describing the experimental program^25,26^, before each test the soil beneath the footing was compacted to its experimentally determined maximum dry density, as obtained from the modified Proctor compaction test, to ensure consistent soil conditions throughout the experimental program. The experimentally measured soil properties were directly incorporated into the validated finite element model, ensuring close agreement between the experimental and numerical investigations. Detailed information regarding the soil characterization, compaction procedure, and material properties is available in the referenced publications. The elastic parameters were assigned as follows: unit weight γ of 18.0, 20.0, and 22.0 kN/m3 for sand, 1:1 sand–crushed stone mixture, and crushed stone, respectively; Young’s modulus (E) of 25,000 MPa, 45,000 MPa, and 65,000 MPa; and Poisson’s ratio (ν) of 0.30, 0.29, and 0.28. The plastic parameters include cohesion (c) as 1, 1 and 1 kPa, internal friction angle φ of 35°, 37°, and 40°, and dilation angle ψ of 5.0°, 7.0°, and 10.0°, respectively. Table 3 illustrates all input soil parameters to fully define the various soil types in this study. These values represent the average measured properties^25,26,32^ and were validated against experimental results to ensure that the numerical model accurately reproduces the observed stress–strain response and failure behavior of the materials.

**Table 3:** Parameters of the soil Mohr-Coulmb material model.

Soil type	Unit weight	Stiffness parameters		Strength parameters			Interface
	${\gamma}_{soil}$ kN/m^3^	${E}_{soil}$ kN/m^2^	${\nu}_{soil}$	C kN/m^2^	Øº	Ψº	${R}_{inter}$
Sand	18.0	25,000	0.30	1	35 º	5 º	0.70
Sand + Crushed Stone (1:1)	20.0	45,000	0.29	1	37 º	7º	0.75
Crushed stone	22.0	65,000	0.28	1	40 º	10 º	0.80

### Interface model

**Fig. 5:**
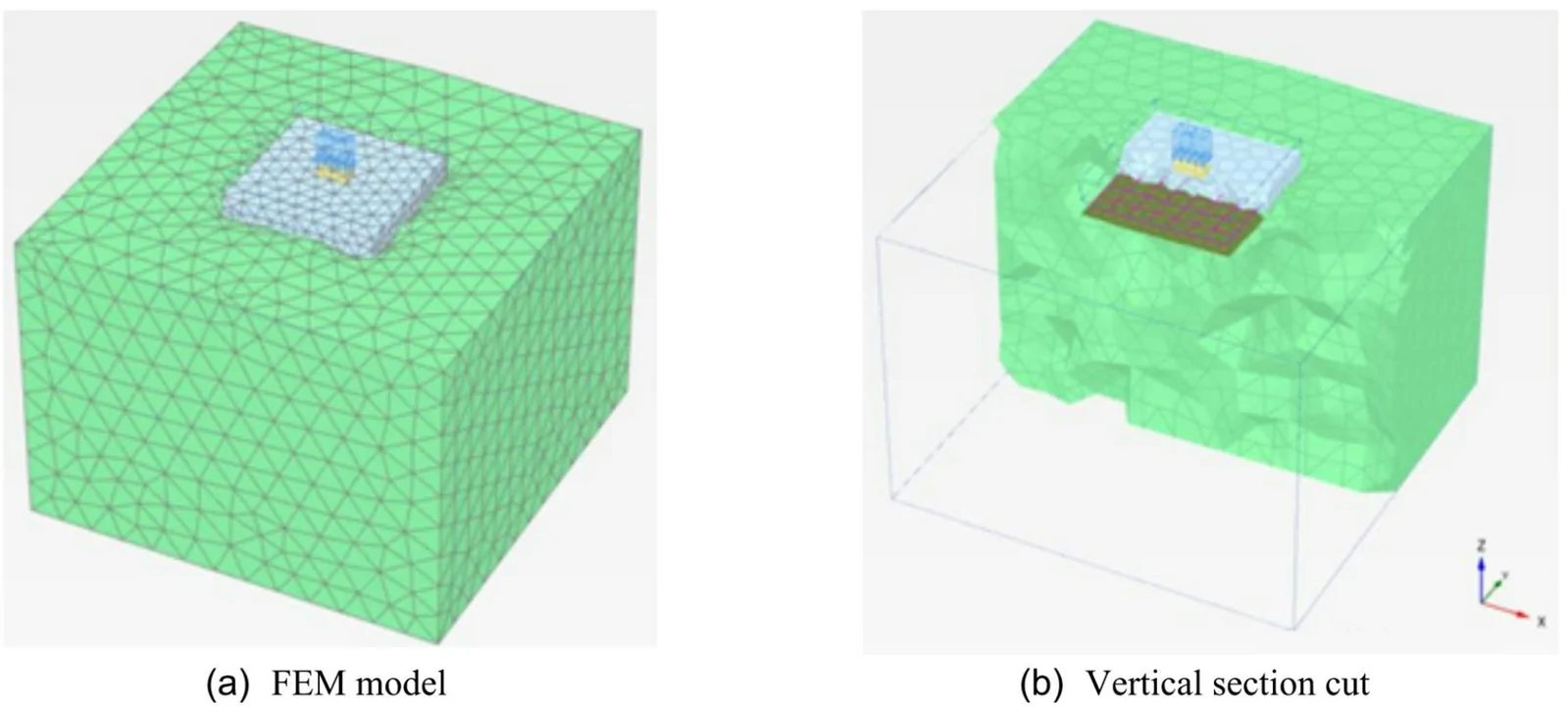
Typical model elements, meshing and applied load.

**Concrete–Soil:** Interface elements were introduced as joint elements connected to the plate elements to realistically simulate the soil–concrete footing interaction. The interface elements consisted of pairs of nodes compatible with the six-node triangular sides of the soil elements or the plate elements, ensuring kinematic compatibility across the soil–structure interface. These interfaces represent a thin zone of concentrated shear deformation at the contact surface between the footing and the surrounding soil. Negative interface elements were assigned along the underside of the footing (local *z*-direction), as illustrated in Fig. 5, to accurately capture contact behavior under applied loading. The interface properties included the adopted constitutive material model and the virtual thickness factor. The interaction between the reinforced concrete footing and the supporting soil was characterized by an appropriate interface strength reduction factor, ${R}_{\mathrm{inter}}$, which relates the interface shear strength to the corresponding soil strength parameters. The applied values of ${R}_{\mathrm{inter}}$ are 0.50, 0.80 and 0.67 for sand, crushed limestone and mixed soil respectively as summarized in Table 3.

**Concrete–Reinforcement:** the reinforcement truss elements were embedded within the host continuum solid elements, as illustrated in Fig. 5. Embedding implies that the translational degrees of freedom at the nodes of the embedded elements are removed and constrained to follow the corresponding interpolated values of the host continuum elements.

### Boundary conditions and loading protocol

A refined mesh was adopted to minimize the effect of mesh dependency on the finite element modeling of cases involving changes in the number and length. The model of the vertically loaded footings on cohesionless soil is set by using the common pattern for the finite element models in geotechnical engineering as the transitional degrees of freedom (u_x_, u_y_, u_z_) were all constrained for all the nodes at the bottom plane of the modeled soil mass. The loading protocol was divided into two main phases to realistically simulate both the laboratory testing procedure and actual footing construction conditions. Usually, the first step in numerical modeling involves defining a geostatic phase that accounts for the initial in-situ stresses and gravity loading. Accordingly, in the initial phase of this study, the soil beneath the tested footing was generated and the geostatic stress state was established. The following loading step is Phase I, the reinforced concrete footing and the loading plate were then introduced, and the application of the main loading stage. Vertical load was applied incrementally through the loading plate and increased step by step until failure occurred, whether in the soil or the reinforced concrete footing, depending on the footing geometry and the properties of the surrounding soil.

### Validation of the developed finite element model

The developed finite element model was validated against the experimental results reported in the authors' previous studies^25,26^. The validation procedure was carried out in three phases to ensure the reliability and accuracy of the numerical model. First, the predicted failure mechanism was compared with the experimentally observed failure mode to verify the compatibility between the numerical and experimental behavior. Second, the incremental load–settlement responses predicted by the finite element model were compared with the corresponding experimental measurements at different locations beneath each footing, including the center, edge, and corner. Finally, the predicted ultimate punching loads were quantitatively compared with the experimental results. The close agreement obtained from these comparisons demonstrated the capability of the developed finite element model to accurately simulate the behavior of reinforced concrete footings resting on soil. Accordingly, the validated finite element model was used to perform the subsequent parametric study and to develop the proposed modification to the current allowable design equation. The applicability of the proposed modified equation is limited to the range of parameters investigated and validated in the present study.

#### Failure mechanism

**Fig. 6:**
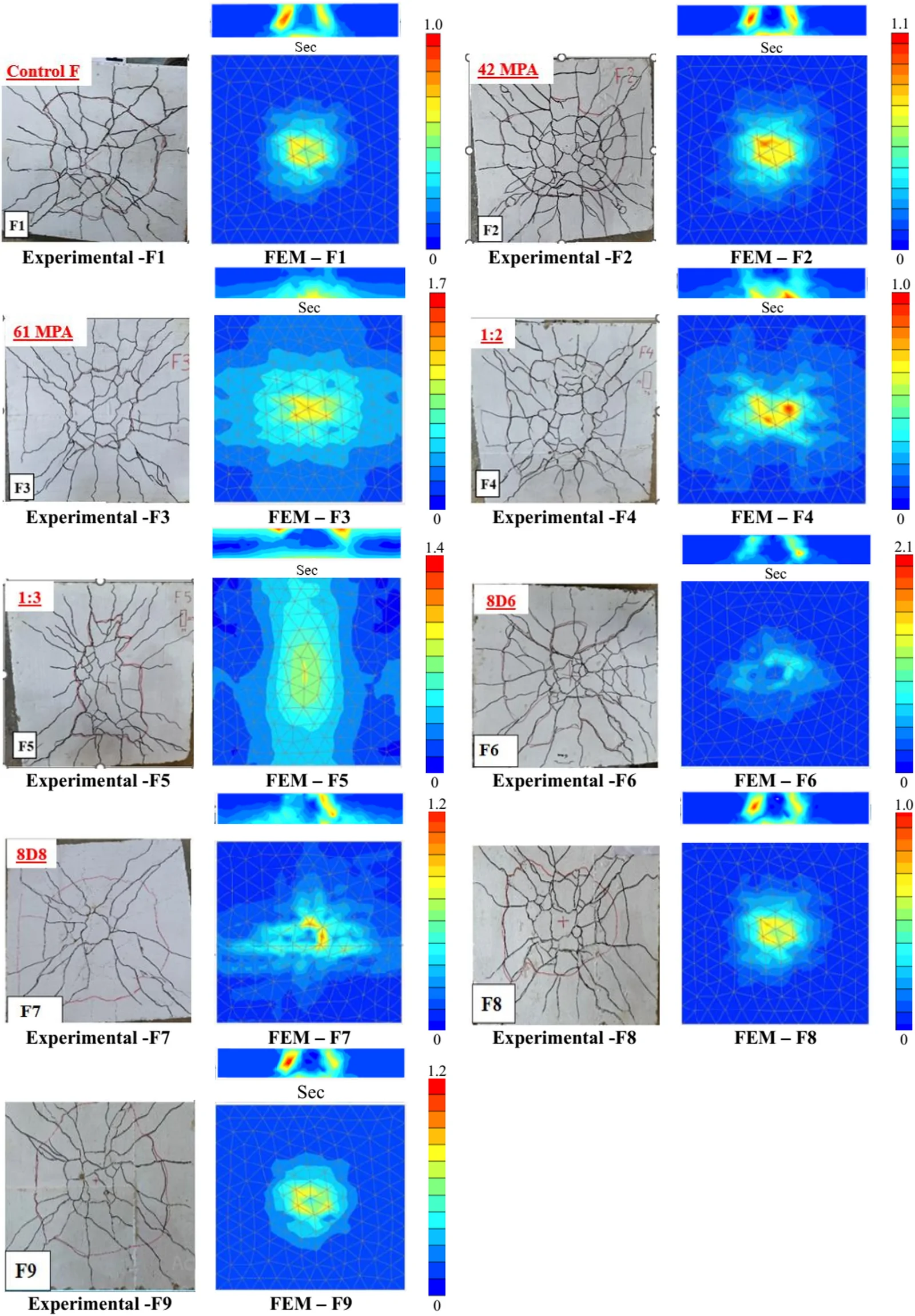
Comparison of Failure Mechanism & shear stress (MPa) of all Tested Footings

**Fig. 7:**
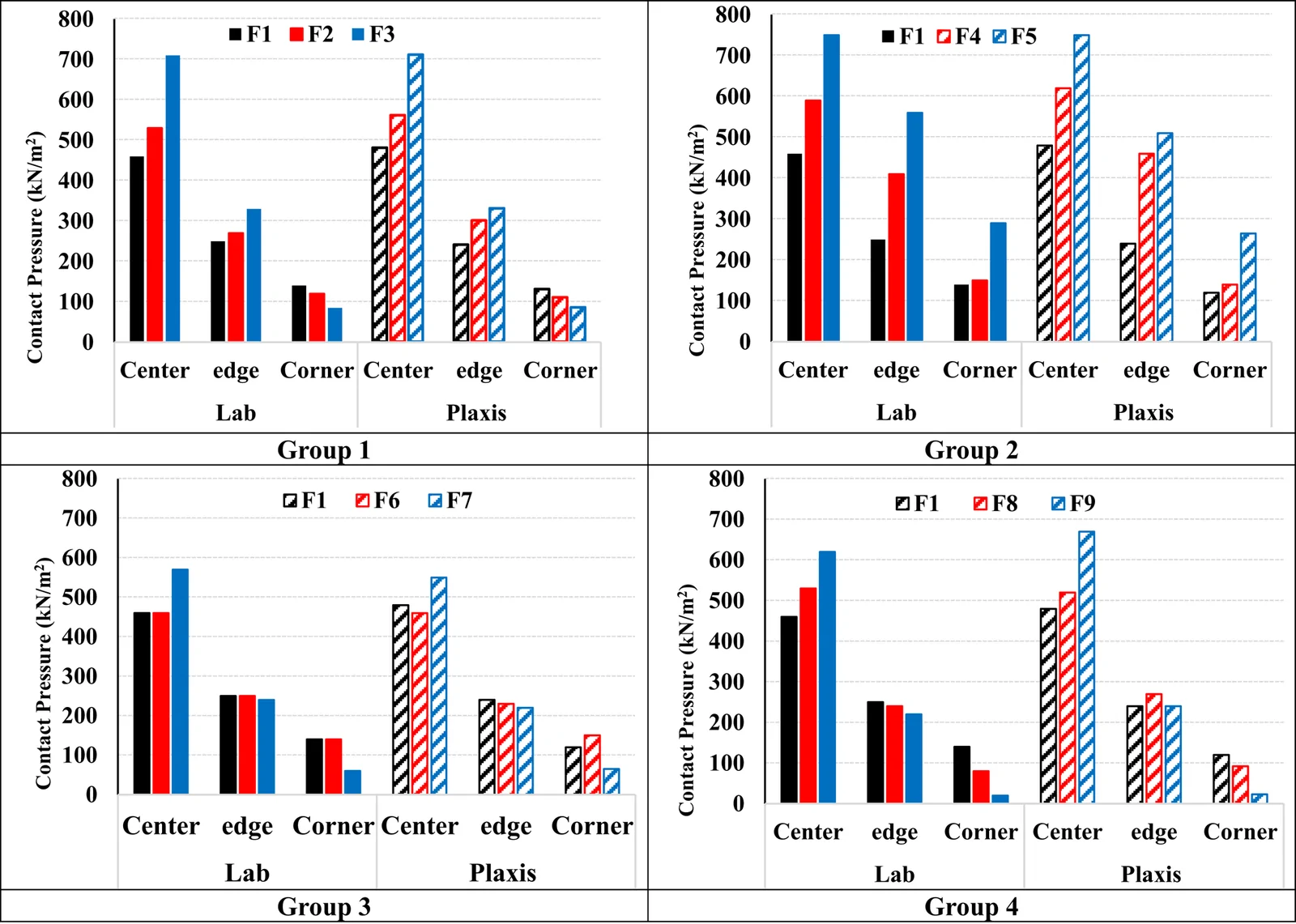
Comparison of contact pressure at different positions(center, edge &corner) below the footing between experimental results and numerical model predictions.

The failure mechanism in the experimental program was identified from the crack patterns observed on the bottom surface of the footings after completion of the loading tests. In the PLAXIS 3D finite element model, the failure mechanism was characterized by the stress distribution at the soil–footing interface, which clearly indicated punching shear failure. For all tested footings (F1 to F9), the maximum principal stresses in the numerical model were consistently concentrated at the bottom surface of the footing, closely corresponding to the locations of experimentally observed cracks. Figure 6 presents a comparison of the failure mechanisms for all tested footings, highlighting the agreement between experimental observations and finite element predictions. Also, the figure below Fig. 7 shows a cross-section of a typical Finite Element Model footing at its center, illustrating the formation of the punching shear cone and the corresponding stress concentration on the footing’s bottom surface, which aligns with the observed cracking pattern.

Contact pressure at failure was evaluated at three representative locations beneath each footing: directly beneath the loading plate, at the mid-edge, and at the corner, as shown in Fig. 7. In all cases, the maximum contact pressure occurred beneath the load center and decreased toward the edges and corners. For Group 1 specimens, the stress contours indicate that the contact pressures at the edge and corner were 54% and 30% of the central value for F1, 51% and 22% for F2, and 43% and 10% for F3, respectively. Increasing the concrete compressive strength resulted in a noticeable rise in contact pressure, with increases of approximately 20% for fcu = 42.5 MPa and up to 100% for fcu = 61 MPa. For Group 2, the relative edge and corner contact pressures were 54% and 30% for F1, 69% and 25% for F4, and 74% and 38% for F5. These results indicate that increasing the column aspect ratio from 1:1 to 1:2 improved the contact pressure distribution by approximately 13.5% and 31.5%, respectively. In Group 3, the corresponding edge and corner contact pressures increased to 79% and 61% for F6 and 91% and 66% for F7. This demonstrates that the inclusion of vertical punching shear reinforcement significantly enhanced stress redistribution, with improvements of approximately 73% for 8Ø6 reinforcement and 84% for 8Ø8 reinforcement. In contrast, for footings rested on the crushed limestone and the mixed sand and crushed stones F8 and F9 footings respectively, the relative contact pressures at the edge and corner decreased to 45% and 15% for F8 and further to 35% and 3% for F9, compared to 54% and 30% for the concentrically loaded footing F1. These findings confirm that increasing soil stiffness beneath the footing leads to stress concentration directly under the applied load, consistent with observations reported in the literature^33^.

As illustrated in Fig. 7, the finite element model accurately reproduces the experimental stress distribution beneath the footing at the center, edge, and corner locations for all investigated groups. A close agreement is observed between laboratory measurements and PLAXIS predictions across Groups 1 to 4, with the Finite Element Model consistently capturing the decreasing trend of contact pressure from the load center toward the edges and corners. Figure 7 summarizes the quantitative comparison of the bar charts indicating that the deviation between numerical and experimental stresses generally remains within ± 2–8% at the footing center, ± 9–13% at the edges, and ± 9–15% at the corners. The slightly larger discrepancies at corner locations are attributed to stress concentration effects, mesh discretization, and localized nonlinear soil behavior near failure. Overall, the observed differences fall within acceptable engineering limits for nonlinear soil–structure interaction modeling, confirming that the developed finite element model provides a reliable representation of contact pressure redistribution beneath reinforced concrete footings up to punching shear failure (Table 4).

**Table 4:** Comparison between the experimental and FEM stress below footings at different positions.

		Experimental (kPa)			FEM (kPa)			FEM/EXP		
Group	Footing	Center	Edge	Corner	Center	Edge	Corner	Center	Edge	Corner
Group 1	F1	460	250	140	480	240	130	1.04	0.96	0.93
	F2	530	270	120	560	300	110	1.06	1.11	0.92
	F3	710	330	85	710	330	85	1.00	1.00	1.00
Group 2	F1	460	250	140	480	240	130	1.04	0.96	0.93
	F4	590	410	150	620	460	140	1.05	1.12	0.93
	F5	750	560	290	750	510	265	1.00	0.91	0.91
Group 3	F1	460	250	140	480	240	130	1.04	0.96	0.93
	F6	460	250	140	460	230	150	1.00	0.92	1.07
	F7	570	240	60	550	220	65	0.96	0.92	1.08
Group 4	F1	460	250	140	480	240	130	1.04	0.96	0.93
	F8	530	240	80	520	270	92	0.98	1.13	1.15
	F9	620	220	20	670	240	23	1.08	1.09	1.15

#### Load-settlement response

**Fig. 8:**
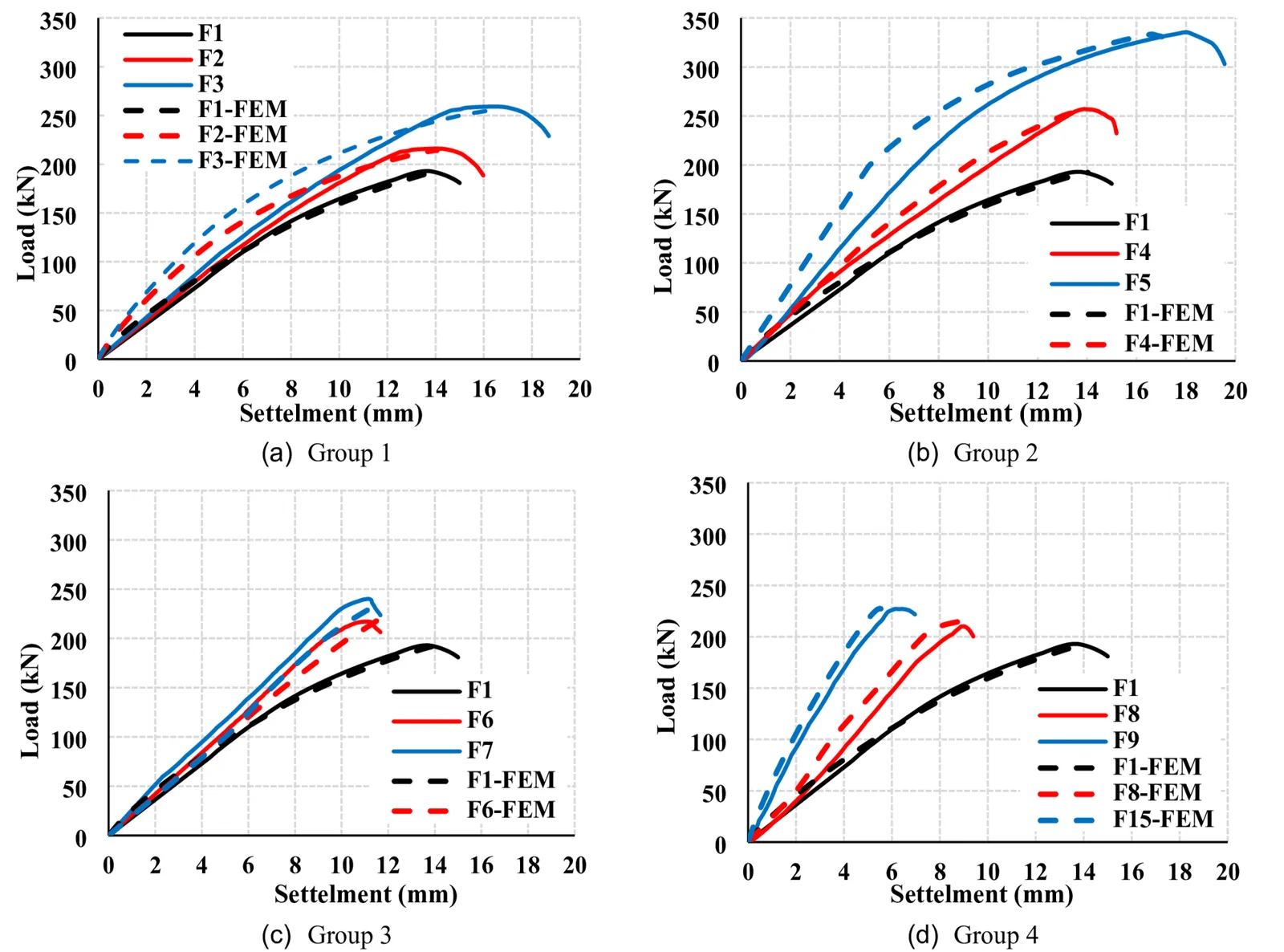
Experimental vs. Finite Element Model (FEM) load-Settlement response for all tested footings.

Validation of the finite element model included comparison with experimental monotonic loading tests on nine footings constructed on identical soil conditions. Load–settlement responses were evaluated at critical locations beneath the footing, including the directly under the loading plate, edge, and corner of the footing to track the variation of the stress distribution and the corresponding influence on the load-settlement response. The response of the footing is governed by the interaction between the reinforced concrete footing and the supporting soil. The load–settlement curves represent the overall behavior of the footing–soil system, where settlement is primarily associated with the progressive deformation of the supporting soil under the applied load. As the load increases, the contact pressure beneath the footing increases, leading to higher stress concentrations within the footing and the initiation and propagation of concrete cracks. Ultimate failure occurs when the punching shear capacity of the footing is reached, while the supporting soil simultaneously experiences significant shear deformation. Consequently, the measured settlement reflects the global soil–structure interaction response, whereas the observed crack pattern characterizes the structural punching shear failure of the reinforced concrete footing. The finite element model captures this coupled behavior by simultaneously simulating the nonlinear response of both the concrete footing and the supporting soil, allowing accurate prediction of the load–settlement response and the corresponding punching shear failure mechanism. The numerical model accurately captured the overall load–settlement behavior at all positions as illustrated in Fig. 8 with settlement discrepancies at the maximum load limited to ± 10% corresponding to low coefficients of variation (0.33%, 0.96%, and 1.11% respectively as summarized in Table 5. Furthermore, the predicted ultimate loads closely matched the experimental values, with a coefficient of variation not exceeding ± 10% as shown in Table 6, demonstrating the reliability of the model for simulating footing–soil interaction under monotonic loading due to the limited scatter which confirm the consistency of the numerical predictions.

**Table 5:** Comparison of experimental and finite element settlement at different locations under each tested footing.

Group No	ID	Experimental settlement			FEM settlement			FEM/EXP		
		Center	Edge	Corner	Center	Edge	Corner	Center	Edge	Corner
		(mm)	(mm)	(mm)	(mm)	(mm)	(mm)			
Control footing	F1	13.67	10.42	9.88	14.00	12.63	12.38	0.98	0.83	0.80
Group 1	F2	14.07	11.32	9.52	14.00	12.60	12.33	1.01	0.90	0.77
	F3	16.49	14.51	13.06	16.80	15.08	14.50	0.98	0.96	0.90
Group 2	F4	13.88	11.81	10.19	14.10	11.93	11.65	0.98	0.99	0.87
	F5	18.00	15.82	16.58	17.00	15.50	15.78	1.06	1.02	1.05
Group 3	F6	11.04	8.84	7.73	11.50	10.08	9.40	0.96	0.88	0.82
	F7	11.17	9.49	8.27	11.80	10.79	10.13	0.95	0.88	0.82
Group 4	F8	8.88	6.63	5.67	8.70	7.20	7.00	1.02	0.92	0.81
	F9	6.23	5.01	4.20	5.50	4.35	4.00	1.13	1.15	1.05
Average								1.01	0.95	0.88
Standard deviation								0.06	0.10	0.11
Variance								0.33%	0.96%	1.11%

**Table 6:** Experimental vs. Finite Element ultimate load for each tested footing.

Group No	Footing ID	Experimental	FEM	FEM/EXP
		Pu	Pu	
		*kN*	*kN*	
Control footing	F1	19.30	19.22	1.00
Group 1	F2	21.60	21.41	1.01
	F3	25.92	25.60	1.01
Group 2	F4	25.70	25.68	1.00
	F5	33.56	33.09	1.01
Group 3	F6	21.70	21.83	0.99
	F7	24.00	23.86	1.01
Group 4	F8	21.00	21.47	0.98
	F9	22.70	22.70	1.00
	Average			1.00
	Standarad deviation			0.01
	Variance			0.01%

## Parametric study

A comprehensive parametric study was conducted to cover all possible combinations of the parameters investigated including f_cu_, column dimensions aspect ratio (a/b), punching shear reinforcement and the soil type. The selected parameter values were adopted from previously published experimental studies and are summarized in Table 7. To account for three different values of each parameter, a total of 81 finite element models (FEM) were developed, representing all possible parameter combinations. These models were used to evaluate the ultimate punching shear capacity predicted by the numerical analysis. In addition, the ultimate punching load (${P}_{u}$), settlement and the subgrade reaction modulus (${K}_{s}$) were determined for each case. All parametric combinations and the corresponding results are provided in the Appendix.

**Table 7:** Applied values for each parameter in parametric study.

Parameter	Applied values			Correction factor
f_cu_ (MPa)	34	42	61	η_1_
col aspect ratio (a/b)	1:1	1:2	1:3	η_2_
Punching reinforcement	None	6 Ø 6	6 Ø 8	η_4_
Soil type	Sand	Sand + crushed limestone	Crushed limestone	η_3_

## Design codes correlation

**Fig. 9:**
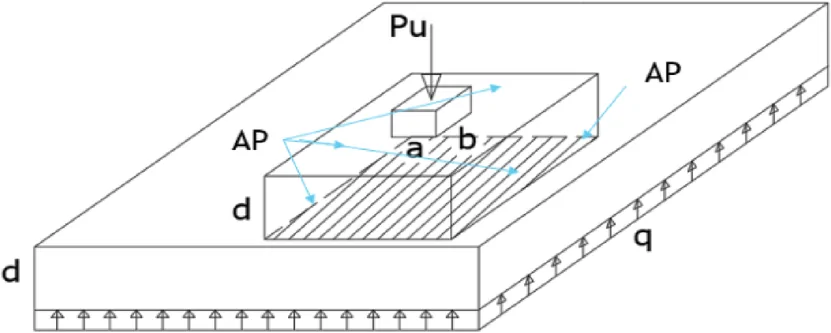
Relationship between predicted and FEM punching shear failure loads.

Current design standards do not provide a dedicated punching shear formulation for reinforced concrete footings resting on soil; instead, available provisions are primarily developed for flat slabs supported by columns as shown in Eq. 1^1,25^. Unlike slab–column systems, footings interact directly with the supporting soil, which generates an upward contact pressure that fundamentally influences the stress distribution, load transfer mechanism, and development of critical shear stresses around the column as provided in Eq. 2. This soil–structure interaction results in a punching shear behavior that differs from that assumed in conventional slab formulations. To enable the application of existing code-based equations to the footing systems, the punching shear provisions of ACI-318^1^ and ECP-203^24^ are adopted in this study with explicit consideration of the upward soil reaction when defining and evaluating the critical shear section. While this adaptation allows the use of current design equations, it does not fully capture the influence of soil stiffness and stress redistribution beneath the footing. To overcome this limitation and enhance the predictive capability of the code equations, the present study introduces modified punching shear expressions incorporating correction factors derived from an extensive parametric finite element investigation as shown in Fig. 9. These correction factors are calibrated within the investigated parameter ranges and are intended to provide a more realistic assessment of the punching shear capacity of reinforced concrete footings subjected to soil–structure interaction effects.

Figure 9 is proposed to predict the ultimate punching shear capacity while explicitly accounting for the key parameters varied in this study, including concrete compressive strength, column dimensions, presence of shear reinforcement, and the ratio of bearing area to footing area. According to ACI-318, the nominal punching shear strength is governed by the concrete compressive strength, the critical perimeter located at a distance of *d/2* from the column face, and the contribution of shear reinforcement, without the inclusion of an explicit size factor. Consequently, the influence of member thickness is considered implicitly within the formulation. The Egyptian Code of Practice (ECP-203)^24^ closely follows the ACI approach, adopting the same critical perimeter definition and similar concrete stress limits, and likewise omits an explicit size effect. For footings with constant thickness, as examined in this study, both ACI^1^ and ECP^24^ therefore yield comparable baseline predictions, with variations in punching shear capacity arising primarily from the other investigated parameters incorporated in Fig. 9.

The proposed correction factors were calibrated using the results of the 81 validated finite element models. The ultimate punching shear capacities predicted by the finite element models are provided in the Appendix, together with the corresponding capacities calculated using the ACI and ECP design equations, which are based on the same punching shear formulation. These datasets were used as the input for the calibration process in Microsoft Excel Solver. Correction factors were introduced as multipliers for the governing variables in the code-based equation, and the optimization process was performed to minimize the differences between the code predictions and the finite element results. including η_1_, η_2_, η₃ and η_4_. Each parameter presents the influence of each parameter of concrete compressive strength, column dimensions aspect ratio, soil-concrete relative stiffness and the shear reinforcement respectively as in traduced in the modified code. The optimized correction factors obtained from the calibration were then incorporated into the modified design equation. The accuracy of the proposed equation is demonstrated in Fig. 9, where the modified equation provides punching shear capacity predictions that closely agree with those obtained from the validated finite element models.

Figure 9 presents the relation between the punching shear capacities predicted by a total of 81 finite element models and those obtained from the code-based design. Among the proposed correction coefficients, η₃ was identified as the dominant influencing factor, while coefficients η₁, η₂, and η₄ remained equal to unity. η₃ was determined to be *ln 5000(ks)* that reflects the soil stiffness major effect. This observation indicates that the applicability of the unmodified design equation is largely restricted to cases involving low-stiffness soils, where the soil pressure distribution beneath the footing tends to be relatively uniform. As soil stiffness increases, however, the resulting non-uniform contact pressure significantly affects the stress concentration near the column and, consequently, the punching shear capacity. In such cases, adjustment of the design equation through the correction factor η₃ becomes necessary to accurately reflect the influence of soil stiffness. Accordingly, η₃ is expressed as a function of the soil subgrade reaction modulus (*ks*), allowing the proposed formulation to capture the transition from uniform to non-uniform stress distributions and their impact on punching shear resistance.

Design codes ultimate punching stress of slabs with shear reinforcement (q_pu_)^1,24^:

1 $${q}_{pu}=0.316\sqrt{{f}_{cu}} . Min(1 or \left(0.50+\frac{a}{b}\right))$$

Ultimate punching stress of footing resting on soil:

2 $${q}_{pu}=\frac{{P}_{u}-q{. A}_{b}}{{A}_{p}}=\frac{{P}_{u} - \frac{{P}_{u}}{A} {. A}_{b}}{{A}_{P}}=\frac{{P}_{u}(1-\frac{{A}_{b}}{A})}{{A}_{P}}$$

Footing Ultimate punching load with shear reinforcement:

3 $${P}_{u}=\frac{{q}_{pu }. {A}_{p}}{1- \frac{{A}_{b}}{A}}+ {A}_{s}{.f}_{y}$$

Substituting Eq. 1 into Eq. 3 yields:

4 $${P}_{u}=\frac{ 0.316\sqrt{{f}_{cu}} . Min\left(1 or \left(0.5+\frac{a}{b}\right)\right).{A}_{p}}{\left(1-\frac{{A}_{b}}{A}\right)}+{A}_{S}.{f}_{y}$$

where; $${q}_{pu }= \frac{{P}_{u}}{{A}_{b}}=\frac{{P}_{u}}{B.L}$$, $${A}_{b}=\left(a+b\right)\left(b+d\right)$$, $${A}_{p}=2.d\left(a+b+2d\right)$$

5 $${\boldsymbol{P}}_{{\boldsymbol{u}}} = \frac{{\eta_{1} 0.316\sqrt {f_{cu} } . \eta_{2} Min\left( {1 , \left( {0.5 + \frac{a}{b}} \right)} \right).A_{p} }}{{\left( {1 - \frac{{A_{b} }}{A}.\eta_{3} } \right)}} + \eta_{4} A_{S} .f_{y}$$

where; ${\upeta}_{1},{\upeta}_{2},{\upeta}_{3}$ are correction factors, ${\mathrm{f}}_{\mathrm{c}\mathrm{u}}$ is the concrete compressive strength, $\mathrm{a}/\mathrm{b}$ is the column aspect ratio, ${\mathrm{A}}_{\mathrm{p}}$ is the critical punching area, ${\mathrm{A}}_{\mathrm{b}}$ represents the bearing area $\mathrm{A}$ is the footing area and ${\upeta}_{3}=\mathrm{l}\mathrm{n} {5000\mathrm{k}}_{\mathrm{s}}$

The proposed modified design equation was developed in two stages. **Stage 1:** Partial correction factors were introduced to account for the effects of concrete compressive strength (${\eta}_{1}$), column aspect ratio ${(\eta }_{2}$) soil stiffness $({\eta}_{3}$), and punching shear reinforcement ${\eta}_{4}$. The values of these correction factors were optimized using the built-in Solver tool in Microsoft Excel to minimize the difference between the finite element predictions (${P}_{u FEM}$) and the values calculated using Eq. 5, (${P}_{u,eq 5}$). The optimized values were 1.00, 1.03, 5.62, and 0.97 for (${\eta}_{1}$), ${(\eta }_{2}$), $({\eta}_{3}$) and ${(\eta }_{4})$ respectively. Since the optimized values of (${\eta}_{1}$), ${(\eta }_{2}$) and ${(\eta }_{4})$ were very close to unity, they were taken as 1.0 in the proposed Eq. 5. The resulting coefficient of determination (R^2^) was 0.925. The optimized value of (${\eta}_{3}$= 5.62) represents the average value obtained from the 81 finite element results as shown in Fig. 9a.

**Stage 2:** The optimized values of (${\eta}_{3}$) were correlated with the soil stiffness (K_s_). Several functional forms were investigated and optimized using the Microsoft Excel Solver tool to minimize the difference between the finite element predictions (${P}_{u,FEM}$) and the values calculated using Eq. 5 (${P}_{u equation 5}$), considering only the soil stiffness correction factor (${\eta}_{3}$). The following relationships were evaluated:

- (${\eta}_{3}$= C,K_s_) : optimized (C = 0.0008), (R^2^ = 0.794) as shown in Fig. 9b
- (${\eta}_{3}$= (C,K_s_)^0.5^): optimized (C = 0.0051), (R^2^ = 0.929) as shown in Fig. 9c
- (${\eta}_{3}$= (C,K_s_) ^0.33^): optimized (C = 0.0309), (R^2^ = 0.966) as shown in Fig. 9d
- (${\eta}_{3}$= ln(C, K_s_) : optimized (C = 0.0544), (R^2^ = 0.973) as shown in Fig. 9e

**Fig. 10:**
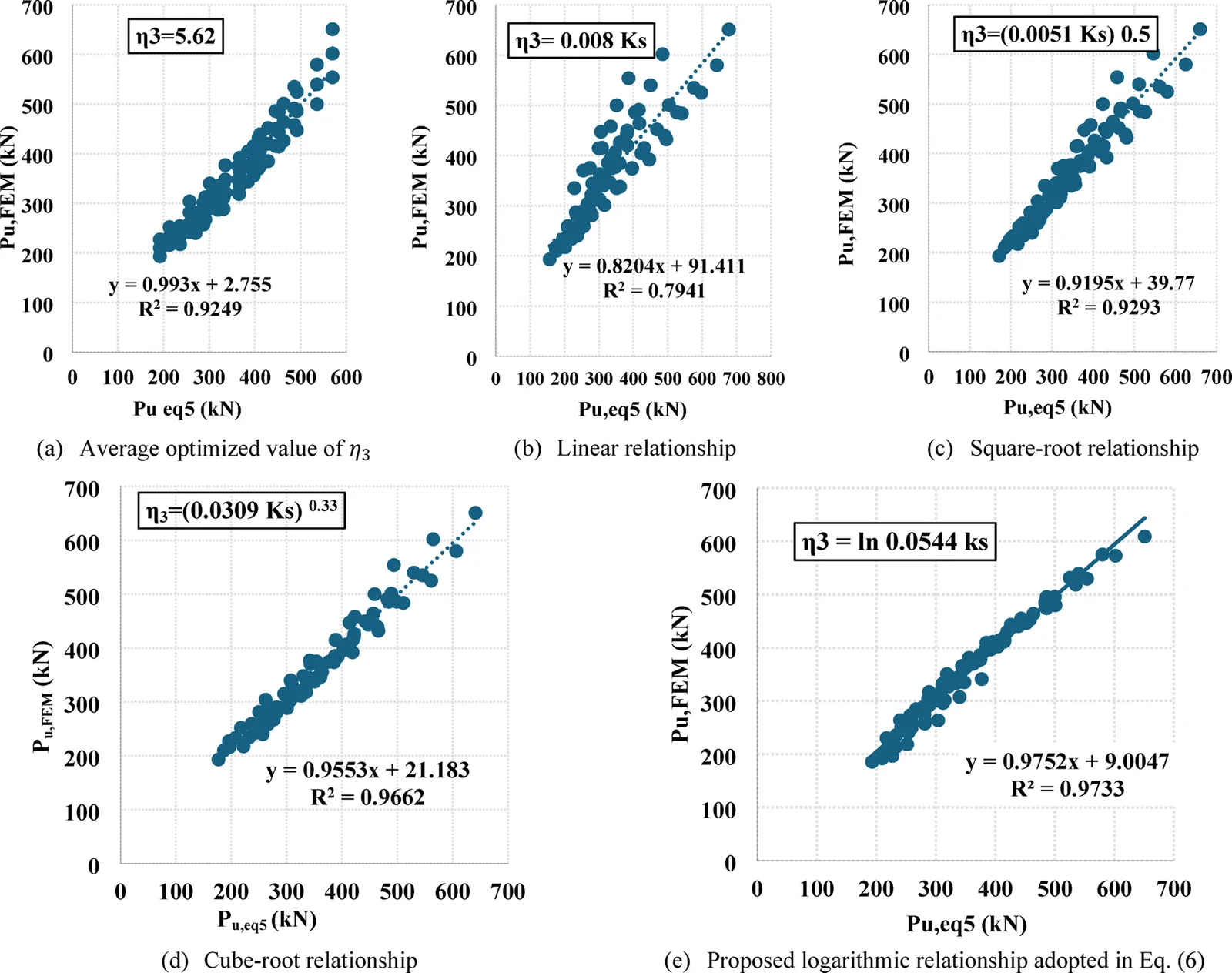
Comparison between the finite element predicted punching load (${P}_{u,\mathrm{F}\mathrm{E}\mathrm{M}}$) and the punching load calculated using Eq. (5) using different formulations of the soil stiffness correction factor (${\eta}_{3}$) investigated during the optimization process.

The logarithmic relationship provided the highest coefficient of determination and was therefore adopted in the proposed design equation. For convenience in practical applications, the coefficient was expressed as ( ln (5000K_s_) ) by expressing (K_s_) in N/mm^3^ and adopting an equivalent practical approximation of the optimized constant. Accordingly, the final form of Eq. 5 was obtained by taking ((${\eta}_{1}=1$), ${(\eta }_{2}=1$) and ${(\eta }_{4}=1)$ and replacing the soil stiffness correction factor with (${\eta}_{3}$ = ln (5000K_s_)) based on the equation units. resulting in the proposed modified design equation, Eq. 6 (Fig. 10).

6 $${{\boldsymbol{P}}}_{{\boldsymbol{u}},{\boldsymbol{m}}{\boldsymbol{o}}{\boldsymbol{d}}{\boldsymbol{i}}{\boldsymbol{f}}{\boldsymbol{i}}{\boldsymbol{e}}{\boldsymbol{d}}}= \frac{0.316\sqrt{{f}_{cu}} . Min\left(1 , \left(0.5+\frac{a}{b}\right)\right).{A}_{p}}{\left(1-\frac{{A}_{b}}{A} . {\boldsymbol{l}}{\boldsymbol{n}}5000{{\boldsymbol{k}}}_{{\boldsymbol{s}}}\right)} +{ A}_{S}.{f}_{y}$$

## Conclusions

This paper proposes a correction to existing code-based punching shear formulas to account for soil–structure interaction effects in RC isolated footings. The correction is derived from validated three-dimensional nonlinear finite element analyses calibrated against experimental test results. A comprehensive parametric study was used to quantify the influence of material properties, geometry, reinforcement, and soil stiffness on punching capacity. The results demonstrate that soil stiffness plays a dominant role in punching shear resistance and is inadequately represented in current slab-based provisions. The proposed correction significantly improves the accuracy and reliability of punching shear predictions for footings resting on soil. This study’s findings can be outlined as follows:

1. The developed three-dimensional nonlinear Finite Element Model accurately reproduced the experimental punching behavior of RC isolated footings, with maximum errors of ± 2% for ultimate load and ± 6% for settlement.
2. Existing design codes (ACI 318 and ECP 203) show noticeable scatter and reduced accuracy, particularly for footings on stiffer soils, as they are generally calibrated for low-stiffness soil conditions with uniform stress distribution.
3. Soil stiffness was identified as a governing factor, significantly increasing punching capacity and subgrade reaction while reducing settlement, highlighting the importance of accounting for soil–structure interaction.
4. Incorporating the proposed soil stiffness correction factor into the code formulas improved prediction accuracy to over 97%, providing a reliable and practical refinement for the punching shear design of RC footings.
5. The applicability of the proposed modified code equation, including the modification factor, is limited to the range of parameters considered in this study, namely concrete compressive strength, column aspect ratio, punching shear reinforcement, and soil stiffness.

## Recommendations for future research

1. Experimental studies are recommended to investigate a wider range of parameters affecting the punching shear behavior of reinforced concrete footings. The resulting data can be used to further verify and enhance both the developed finite element model and the proposed modified code equation.
2. The developed finite element model should be verified against experimental results covering a broader range of the parameters investigated in this study, including concrete compressive strength, column aspect ratio, punching shear reinforcement, and loading eccentricity.
3. The finite element model should also be validated for additional parameters that were not considered in the present study, such as footing geometry, column geometry, soil conditions, reinforcement ratio, and loading configurations, to assess its general applicability.
4. The proposed modified code equation should be validated and, if necessary, refined using the results of the recommended experimental and numerical investigations to confirm its applicability over a wider range of structural and geotechnical conditions.

## Supplementary Information

Below is the link to the electronic supplementary material.

Supplementary Material 1

## Data Availability

The data that supports the findings of this study are available in the paper text.

## Abbreviations

A: Footing area
A_b_: Bearing area
A_p_: Critical punching shear area
a: Column dimension in one direction
b: Column dimension in the perpendicular direction
a/b: Column aspect ratio
c: Soil cohesion
d: Effective depth
E: Young's modulus
E_28_: Young's modulus at 28 days
f_c,28_: Concrete compressive strength at 28 days
f_ck_: Characteristic concrete compressive strength
f_cu_: Concrete cube compressive strength
f_t,28_: Concrete tensile strength at 28 days
F_c_: Concrete compressive strength
G_c_: Compressive fracture energy
G_t_: Tensile fracture energy
H_c_: Normalized hardening–softening parameter
k_s_: Modulus of subgrade reaction
P_u_: Ultimate punching load
R_inter_: Interface strength reduction factor
V_c_: Concrete punching shear capacity
V_n_: Nominal punching shear strength
γ: Unit weight
γ_fc_: Safety factor for concrete compressive strength
γ_ft_: Safety factor for concrete tensile strength
η_1_: Concrete strength correction factor
η_2_: Column aspect ratio correction factor
η_3_: Soil stiffness correction factor
η_4_: Shear Reinforcement correction factor
ν: Poisson's ratio
φ: Internal friction angle
φ_max_: Maximum friction angle
ψ: Soil dilation angle
Ψ: Concrete dilation angle
σ_y_: Steel yield strength
ε_p3_: Minor plastic strain
ε_pcp_: Plastic strain at peak compressive stress
Ø: Reinforcement bar diameter
ACI: American concrete institute
CDP: Concrete damaged plasticity
ECP: Egyptian code of practice
FE: Finite element
FEM: Finite element model
MC: Mohr–coulomb
PLAXIS: PLAXIS 3D finite element software
RC: Reinforced concrete
SSI: Soil–structure interaction
