# Supplementary material for: Code-based refinement and finite element validation of punching shear design for reinforced concrete footings
Source: Sci Rep. 2026 Sep 9;16:28240. doi: 10.1038/s41598-026-68850-7 (PMC13558554; doi:10.1038/s41598-026-68850-7)
Supplement: Supplementary file 1 [file 41598_2026_68850_MOESM1_ESM.docx]

APPENDIX

| **Model No.** | **f_cu_ Mpa** | **b/a** | **Punching RFT** | **Soil Type** | **FEM**  **P_u_ kN** | **Settlement (mm)** | **Ks (kN/m^3^)** |
| --- | --- | --- | --- | --- | --- | --- | --- |
| **1** | 34 | 1 | None | Sand | 192 | 13.67 | 28833 |
| **2** | 34 | 1 | None | Sand & Crushed Limestone | 214 | 8.7 | 50496 |
| **3** | 34 | 1 | None | Crushed Limestone | 227 | 5.5 | 84727 |
| **4** | 34 | 1 | 8D6 | Sand | 218 | 11.04 | 40537 |
| **5** | 34 | 1 | 8D6 | Sand & Crushed Limestone | 234 | 8.83 | 54402 |
| **6** | 34 | 1 | 8D6 | Crushed Limestone | 254 | 5.74 | 90841 |
| **7** | 34 | 1 | 8D8 | Sand | 240 | 11.17 | 44108 |
| **8** | 34 | 1 | 8D8 | Sand & Crushed Limestone | 259 | 9.15 | 58108 |
| **9** | 34 | 1 | 8D8 | Crushed Limestone | 280.8 | 5.8 | 99387 |
| **10** | 34 | 2 | None | Sand | 256 | 14.02 | 37484 |
| **11** | 34 | 2 | None | Sand & Crushed Limestone | 279 | 11.49 | 49848 |
| **12** | 34 | 2 | None | Crushed Limestone | 301.32 | 7.29 | 84852 |
| **13** | 34 | 2 | 8D6 | Sand | 288.61 | 14.72 | 40250 |
| **14** | 34 | 2 | 8D6 | Sand & Crushed Limestone | 311.22 | 12.07 | 52932 |
| **15** | 34 | 2 | 8D6 | Crushed Limestone | 337.82 | 7.65 | 90653 |
| **16** | 34 | 2 | 8D8 | Sand | 318.68 | 14.9 | 43906 |
| **17** | 34 | 2 | 8D8 | Sand & Crushed Limestone | 345.96 | 12.21 | 58166 |
| **18** | 34 | 2 | 8D8 | Crushed Limestone | 373.6 | 7.74 | 99089 |
| **19** | 34 | 3 | None | Sand | 330 | 17 | 39850 |
| **20** | 34 | 3 | None | Sand & Crushed Limestone | 362.7 | 13.94 | 53413 |
| **21** | 34 | 3 | None | Crushed Limestone | 392 | 8.84 | 91032 |
| **22** | 34 | 3 | 8D6 | Sand | 375.2 | 17.77 | 43345 |
| **23** | 34 | 3 | 8D6 | Sand & Crushed Limestone | 406.22 | 14.2 | 58726 |
| **24** | 34 | 3 | 8D6 | Crushed Limestone | 439 | 9.24 | 97533 |
| **25** | 34 | 3 | 8D8 | Sand | 415.4 | 18.07 | 47192 |
| **26** | 34 | 3 | 8D8 | Sand & Crushed Limestone | 449.7 | 14.81 | 62334 |
| **27** | 34 | 3 | 88D | Crushed Limestone | 486 | 9.4 | 106137 |
| **28** | 42 | 1 | None | Sand | 214.1 | 14.05 | 31282 |
| **29** | 42 | 1 | None | Sand & Crushed Limestone | 233 | 11.52 | 41521 |
| **30** | 42 | 1 | None | Crushed Limestone | 251.9 | 7.3 | 70838 |
| **31** | 42 | 1 | 8D6 | Sand | 241.92 | 14.75 | 33670 |
| **32** | 42 | 1 | 8D6 | Sand & Crushed Limestone | 260 | 12.09 | 44148 |
| **33** | 42 | 1 | 8D6 | Crushed Limestone | 282 | 7.67 | 75477 |
| **34** | 42 | 1 | 8D8 | Sand | 267 | 14.93 | 36712 |
| **35** | 42 | 1 | 8D8 | Sand & Crushed Limestone | 288 | 12.24 | 48303 |
| **36** | 42 | 1 | 88D | Crushed Limestone | 312.3 | 7.76 | 82617 |
| **37** | 42 | 2 | None | Sand | 287 | 18.68 | 31540 |
| **38** | 42 | 2 | None | Sand & Crushed Limestone | 310 | 15.31 | 41567 |
| **39** | 42 | 2 | None | Crushed Limestone | 335 | 9.7 | 70898 |
| **40** | 42 | 2 | 8D6 | Sand | 321.4 | 19.61 | 33646 |
| **41** | 42 | 2 | 8D6 | Sand & Crushed Limestone | 347.2 | 16.08 | 44325 |
| **42** | 42 | 2 | 8D6 | Crushed Limestone | 375 | 10.2 | 75473 |
| **43** | 42 | 2 | 8D8 | Sand | 355.8 | 19.85 | 36796 |
| **44** | 42 | 2 | 8D8 | Sand & Crushed Limestone | 384 | 16.3 | 48362 |
| **45** | 42 | 2 | 88D | Crushed Limestone | 415 | 10.32 | 82552 |
| **46** | 42 | 3 | None | Sand | 370 | 22.62 | 33579 |
| **47** | 42 | 3 | None | Sand & Crushed Limestone | 396 | 18.54 | 43848 |
| **48** | 42 | 3 | None | Crushed Limestone | 432 | 11.7 | 75798 |
| **49** | 42 | 3 | 8D6 | Sand | 414.4 | 23.751 | 35818 |
| **50** | 42 | 3 | 8D6 | Sand & Crushed Limestone | 443.5 | 19.47 | 46761 |
| **51** | 42 | 3 | 8D6 | Crushed Limestone | 483.8 | 12.3 | 80746 |
| **52** | 42 | 3 | 8D8 | Sand | 458 | 24.04 | 39110 |
| **53** | 42 | 3 | 8D8 | Sand & Crushed Limestone | 491 | 19.7 | 51165 |
| **54** | 42 | 3 | 88D | Crushed Limestone | 535 | 12.5 | 87862 |
| **55** | 61 | 1 | None | Sand | 256 | 16.6 | 31659 |
| **56** | 61 | 1 | None | Sand & Crushed Limestone | 281.4 | 13.6 | 42476 |
| **57** | 61 | 1 | None | Crushed Limestone | 304 | 8.63 | 72314 |
| **58** | 61 | 1 | 8D6 | Sand | 290 | 17.43 | 34155 |
| **59** | 61 | 1 | 8D6 | Sand & Crushed Limestone | 315 | 14.29 | 45252 |
| **60** | 61 | 1 | 8D6 | Crushed Limestone | 340 | 9.06 | 77039 |
| **61** | 61 | 1 | 8D8 | Sand | 321.4 | 17.64 | 37403 |
| **62** | 61 | 1 | 8D8 | Sand & Crushed Limestone | 348 | 14.46 | 49405 |
| **63** | 61 | 1 | 88D | Crushed Limestone | 377 | 9.17 | 84398 |
| **64** | 61 | 2 | None | Sand | 344 | 22.15 | 31882 |
| **65** | 61 | 2 | None | Sand & Crushed Limestone | 374.6 | 18.16 | 42346 |
| **66** | 61 | 2 | None | Crushed Limestone | 404.32 | 11.51 | 72112 |
| **67** | 61 | 2 | 8D6 | Sand | 385 | 23.25 | 33994 |
| **68** | 61 | 2 | 8D6 | Sand & Crushed Limestone | 420 | 19.06 | 45236 |
| **69** | 61 | 2 | 8D6 | Crushed Limestone | 452 | 12.09 | 76749 |
| **70** | 61 | 2 | 8D8 | Sand | 426 | 23.54 | 37150 |
| **71** | 61 | 2 | 8D8 | Sand & Crushed Limestone | 464 | 19.3 | 49354 |
| **72** | 61 | 2 | 88D | Crushed Limestone | 501 | 12.24 | 84026 |
| **73** | 61 | 3 | None | Sand | 447.2 | 26.86 | 34179 |
| **74** | 61 | 3 | None | Sand & Crushed Limestone | 486 | 22.02 | 45308 |
| **75** | 61 | 3 | None | Crushed Limestone | 525 | 14 | 76982 |
| **76** | 61 | 3 | 8D6 | Sand | 500 | 28.2 | 36398 |
| **77** | 61 | 3 | 8D6 | Sand & Crushed Limestone | 540 | 23.12 | 47947 |
| **78** | 61 | 3 | 8D6 | Crushed Limestone | 580 | 14.66 | 81218 |
| **79** | 61 | 3 | 8D8 | Sand | 554 | 28.55 | 39835 |
| **80** | 61 | 3 | 8D8 | Sand & Crushed Limestone | 602 | 23.41 | 52790 |
| **81** | 61 | 3 | 88D | Crushed Limestone | 651 | 14.84 | 90055 |
